# Recent developments in translational imaging of *in vivo* gene therapy outcomes

**DOI:** 10.1016/j.ymthe.2024.12.049

**Published:** 2024-12-30

**Authors:** Isabel L. Day, Mikayla Tamboline, Gerald S. Lipshutz, Shili Xu

**Affiliations:** 1 Department of Molecular and Medical Pharmacology, David Geffen School of Medicine, University of California, Los Angeles, Los Angeles, CA 90095, USA; 2 Crump Institute for Molecular Imaging, University of California, Los Angeles, Los Angeles, CA 90095, USA; 3 Department of Psychiatry and Biobehavioral Sciences, David Geffen School of Medicine, University of California, Los Angeles, Los Angeles, CA 90095, USA; 4 Department of Surgery, David Geffen School of Medicine, University of California, Los Angeles, Los Angeles, CA 90095, USA; 5 Intellectual and Developmental Disabilities Research Center, University of California, Los Angeles, Los Angeles, CA 90095, USA; 6 Semel Institute for Neuroscience, University of California, Los Angeles, Los Angeles, CA 90095, USA; 7 Molecular Biology Institute, David Geffen School of Medicine, University of California, Los Angeles, Los Angeles, CA 90095, USA; 8 Jonsson Comprehensive Cancer Center, University of California, Los Angeles, Los Angeles, CA 90095, USA

**Keywords:** gene therapy, imaging, biomarker, therapeutic outcome, cancer, metabolic disorders, degenerative disorders

## Abstract

Gene therapy achieves therapeutic benefits by delivering genetic materials, packaged within a delivery vehicle, to target cells with defective genes. This approach has shown promise in treating various conditions, including cancer, metabolic disorders, and tissue-degenerative diseases. Over the past 5 years, molecular imaging has increasingly supported gene therapy development in both preclinical and clinical studies. High-quality images from positron emission tomography (PET), single-photon emission computed tomography (SPECT), magnetic resonance imaging (MRI), and computed tomography (CT) enable quantitative and reliable monitoring of gene therapy. Most reported studies have applied imaging biomarkers to non-invasively evaluate the outcomes of gene therapy. This review aims to inform researchers in molecular imaging and gene therapy about the integration of these two disciplines. We highlight recent developments in using imaging biomarkers to monitor the outcome of *in vivo* gene therapy, where the therapeutic delivery vehicle is administered systemically. In addition, we discuss prospects for further incorporating imaging biomarkers to support the development and application of gene therapy.

## Graphical abstract

**Figure:**
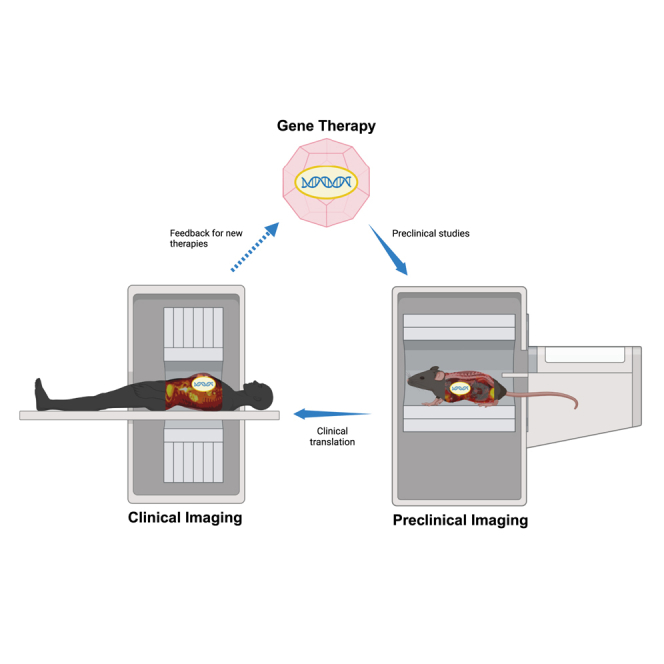

## Teaser

Xu and colleagues present recent advances in using imaging biomarkers to monitor *in vivo* gene therapy in cancer and metabolic and tissue-degenerative diseases and explore potential opportunities to further integrate these biomarkers to overcome current challenges in gene therapy, aiming to inspire interdisciplinary collaboration in imaging and gene therapy.

## Introduction

Gene therapy was first proposed in 1972 by physician Theodore Friedmann and biochemist Richard Roblin.^1^ In their groundbreaking paper, they introduced the revolutionary idea of delivering genes into specific cell types to treat human diseases by modifying host DNA. Unlike conventional therapies, gene therapy utilizes nucleic acids or genes as the therapeutic agent, which are packaged within a delivery vehicle to target and enter the cells with defective genes. Once inside the target cells, the therapeutic nucleic acids can modify DNA and genetic function, leading to treatment or significant improvements in patients’ quality of life.^2^

Gene therapy strategies encompass gene editing, gene replacement, gene addition, and gene inhibition. Depending on the approach, gene therapies are categorized as *in vivo* gene therapy and *ex vivo* gene therapy.^3^ This review focuses on *in vivo* gene therapy, where the therapeutic delivery vehicle is administered systemically into the patient’s blood circulation or cerebrospinal fluid. Viral vectors, such as adeno-associated virus (AAV) vectors, are most commonly used for *in vivo* gene therapy delivery. In certain cases, *in vivo* gene therapy vehicles are administered directly to a specific organ or region in the patient, a process sometimes referred to as *in situ* gene therapy.^4^ In contrast, *ex vivo* gene therapy involves removing target cells from the patient, genetically engineering them, and then re-infusing them back into the patient to achieve a therapeutic effect. This approach, which also sometimes falls into the category of cell therapy, is particularly applicable to blood diseases.

After five decades of research and development, *in vivo* gene therapy has emerged as a treatment option for a wide range of diseases, including cancer, metabolic disorders, and tissue-degenerative diseases.^5^ *In vivo* gene therapy for cancer aims to control altered genes or genetic mutations in cancer cells to inhibit cancer progression or induce direct cancer cell killing. By using tumor-specific promoters, the therapeutic genes turn on their localized activity within the tumor.^6^ Gene therapies for metabolic disorders and tissue-degenerative disease are providing new hope to patients and their families. Treatments for Parkinson’s disease,^7^^,^^8^^,^^9^^,^^10^ refractory epilepsies,^11^ and sphingolipidoses^12^ have advanced to clinical trials in recent years. These early advancements highlight the significant potential of *in vivo* gene therapy to address complex medical conditions.

**Figure 1:**
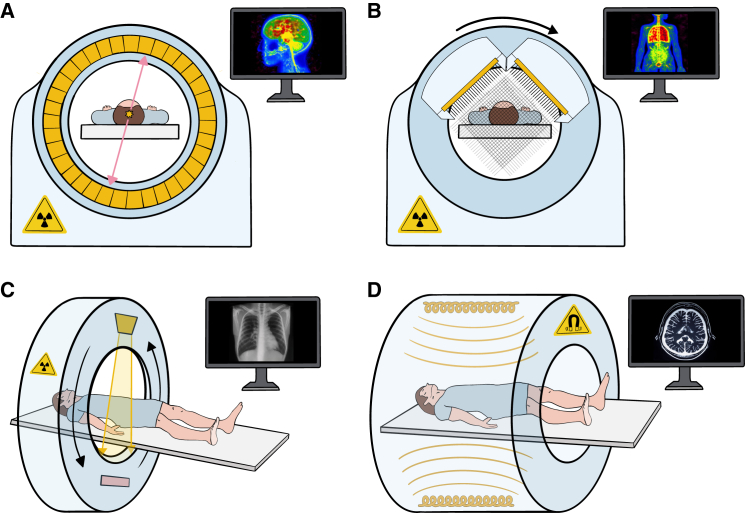
Translational imaging modalities for non-invasive imaging of gene therapy outcomes Gene therapy outcomes can be monitored using translational imaging modalities: (A) positron emission tomography (PET), (B) single-photon emission computed tomography (SPECT), (C) computed tomography (CT), and (D) magnetic resonance imaging (MRI).

The advancement of gene therapy is supported by the development and application of powerful molecular imaging tools. These tools enable the visualization of various aspects of gene therapy, including vehicle delivery,^13^ therapeutic gene expressing location and duration, and therapeutic efficacy,^14^ through the use of suitable imaging biomarkers. Major methods for non-invasive imaging of *in vivo* gene therapy outcomes include positron emission tomography (PET), single-photon emission computed tomography (SPECT), computed tomography (CT), and magnetic resonance imaging (MRI) (Figure 1). Among these imaging modalities, PET and SPECT are functional imaging techniques harnessed to probe biological processes and activities *in vivo*. PET imaging involves the injection of PET probes containing positron-emitting radionuclides, such as F-18, Ga-68, Cu-64, and Zr-89, into patients. A PET scanner detects coincidence of colinear 511 keV photon pairs that result from positron-electron annihilation within several millimeters of the positron emitter.^15^ SPECT imaging, based on the detection of single gamma rays, requires the injection of imaging probes containing gamma-emitting nuclides, such as Tc-99m, I-123, I-131, and In-111, into patients.^16^ Compared with SPECT scans, PET scans are faster, offer better image resolution and clearer details, and require lower levels of radioactivity, making PET scans more common for monitoring gene therapy outcomes. Unlike PET or SPECT, MRI and CT scans provide anatomic information, such as changes in tissue composition and structure that occur during and after gene therapy. The principle of CT imaging is based on the attenuation of an X-ray beam as it passes through tissue, which is proportional to the tissue’s electron density, generally aligned with its physical density. Therefore, CT is widely used to visualize bone structures, while MRI provides superior contrast for soft tissues, such as the brain and muscle. Overall, these imaging modalities play a crucial role in advancing the field of gene therapy by enabling precise monitoring and assessment of therapeutic interventions.

Distinguishing between different types of imaging biomarkers is essential, as outlined by Sellmyer et al. in their description of a clinical imaging paradigm of predictive, therapeutic, and pharmacodynamic biomarkers.^17^ Predictive biomarkers determine whether a therapeutic target is present and monitor its expression before administering gene therapy. Therapeutic biomarkers evaluate whether the therapy successfully reached its target. Pharmacodynamic biomarkers measure therapeutic outcomes by tracking biochemical processes or phenotypic changes that occur, often downstream of the therapy’s target. This review emphasizes the therapeutic outcomes of gene therapies, focusing primarily on pharmacodynamic biomarkers. However, these categories are not always mutually exclusive. For instance, the reporter genes herpes simplex virus type 1 thymidine kinase (HSV1-TK) and sodium iodide symporter (NIS), discussed below, are generally classified as therapeutic biomarkers due to their role in tracking gene therapy by being encoded directly within the therapeutic vector. Yet, these same reporter genes can also assess gene therapy outcomes in cancer. Through coupling with PET or SPECT radiotracers, they enable monitoring of the activity or uptake of radiolabeled substrates in tumor cells—both key biochemical indicators of cancer cell response to gene therapy. Because of this versatility, these imaging reporter examples are included in this review.

In this review, we explore the recent advancements and applications of molecular imaging methods, including PET, SPECT, MRI, and CT, in monitoring biomarkers that indicate gene therapy outcomes. The integration of these powerful imaging tools not only enhances our understanding of gene therapy mechanisms in preclinical models but also holds significant potential for clinical translation.

## Translational imaging of gene therapy outcomes in cancer

In recent years, gene therapy has broadened its scope beyond solely addressing single-gene genetic disorders to include complex genetic and inherited conditions, such as cancer.^18^^,^^19^ Various gene therapy methods have been developed for cancer management, with *in vivo* methods including gene-directed-enzyme prodrug therapy (GDEPT) and radionuclide gene therapy.^18^^,^^20^^,^^21^ Due to its established history in cancer diagnosis and tracking disease progression, molecular imaging has emerged as a crucial tool for non-invasively monitoring gene therapy outcomes for cancer.^22^^,^^23^^,^^24^ Therapeutic and pharmacodynamic imaging biomarkers have been extensively quantified using PET and SPECT to evaluate the efficacy of gene therapy methods in cancer management.^17^^,^^25^ An overview of applications of translational imaging in gene therapy to treat cancer is included in Table 1.

**Table 1:** Application of translational imaging in gene therapy to treat cancer

Biomarker	Tracer	Imaging modality	References
HSV1-TK	^18^F-FHBG	PET	Lee et al.^27^; Sekar et al.^28^; Liu et al.^32^; Lee et al.^33^; Yaghoubi et al.^34^; Salabert et al.^35^; Kuruppu et al.^36^; Li et al.^43^; Yaghoubi et al.^46^; Keu et al.^47^; Yaghoubi et al.^78^
	^124^I-FIAU	PET	Seo et al.^38^
	^123^I-FIAU	SPECT	Wang et al.^31^; Choi et al.^37^; Dempsey et al.^40^
	^18^F-FUdR	PET	Wang et al.^31^
	^18^F-FPCV	PET	Cai et al.^39^
	^18^F-FEAU	PET	Miyagawa et al.^44^
NIS	^18^F-TFB	PET	Ahn et al.^48^; Samnick et al.^56^; Dittmann et al.^57^; Ventura et al.^58^; O’Doherty et al.^66^; Diocou et al.^67^; Jiang et al.^68^
	^124^I-NaI	PET	Kitzberger et al.^20^; Lee et al.^27^; Ahn et al.^48^; Kitzberger et al.^51^; Schmohl et al.^53^; Samnick et al.^56^
	^123^I-NaI	SPECT	Ahn et al.^48^; Carlson et al.^59^; Penheiter et al.^61^
	^125^I-NaI	SPECT	Zhang et al.^60^; Shi et al.^62^; Marsee et al.^63^; Zhang et al.^64^
	^99m^Tc-MAA	SPECT	Marsee et al.^63^
	Na^99m^TcO_4_	SPECT	Marsee et al.^63^
	Na^188^ReO_4_	SPECT	Zhang et al.^64^
eDHFR	^11^C-TMP	PET	Sellmyer et al.^71^
	^18^F-TMP	PET	Sellmyer et al.^72^; Lee et al.^73^
Glycolysis	^18^F-FDG	PET	Sellmyer et al.^17^; Wang et al.^31^; Dittmann et al.^57^; Diocou et al.^67^; Schwenck et al.^75^; Yaghoubi et al.^78^; Lee et al.^88^
L-amino acid transporter	^18^F-FET	PET	Wang et al.^31^; Schwenck et al.^75^; Nagy et al.^79^; Waltenberger et al.^80^; Manzarbeitia-Arroba et al.^81^; Harat et al.^82^
Tumor cell proliferation	^18^F-FLT	PET	Bashir et al.^83^; Rainone et al.^84^; Kairemo et al.^85^

### Imaging therapeutic biomarkers for cancer gene therapy outcomes

#### HSV1-TK as a theranostic imaging reporter gene

In cancer imaging, therapeutic biomarkers refer to those that assess whether a therapy has successfully reached a target, including imaging reporter genes.^17^^,^^26^ Certain reporter genes introduced by gene therapy can be used to evaluate the efficacy of cancer gene therapy treatment, thus serving a dual role in tracking gene delivery while assessing gene therapy outcomes.^27^ One example that has been used most extensively for cancer gene therapy and translational imaging as a theranostic reporter gene is HSV1-TK.^21^^,^^27^^,^^28^^,^^29^^,^^30^^,^^31^^,^^32^^,^^33^^,^^34^^,^^35^^,^^36^^,^^37^^,^^38^^,^^39^^,^^40^ The mechanism of HSV1-TK gene therapy involves transfer of the HSV1-TK gene to tumor cells using *in vivo* delivery methods, which, in turn, allows for GDEPT and direct imaging of cancer cells with active HSV1-TK expression.^41^ HSV1-TK has been widely explored as a gene therapy agent for cancer.^21^^,^^29^^,^^40^^,^^41^^,^^42^^,^^43^

Various radiotracers have been applied to assess tumor responses to HSV1-TK gene therapy using PET and SPECT imaging, some more successfully than others.^28^^,^^31^^,^^32^^,^^33^^,^^35^^,^^36^^,^^37^^,^^38^^,^^39^^,^^44^ An early preclinical study by Wang et al. compared the potential of ^123^I-5-iodo-2′-fluoro-1-β-D-arabinofuranosyluracil (^123^I-FIAU), 5-^18^F-fluoro-2′-deoxyuridine (^18^F-FUdR), 2-^18^F-fluoroethyl-L-tyrosine (^18^F-FET), and ^18^F-fluoro-2-deoxyglucose (^18^F-FDG) for their ability to monitor HSV1-TK expression, concluding that ^123^I-FIAU was the most reliable method for monitoring tumor response to GDEPT.^31^ Other FIAU PET radiotracers have since demonstrated success in monitoring GDEPT outcomes in preclinical settings, most notably ^124^I-2′-fluoro-2′-deoxy-1-β-D-arabinofuranosyl-5-iodouracil.^37^^,^^38^ However, the popularity of FIAU radiotracers has recently declined due to limited success in clinical studies^40^ and the development of improved imaging agents. The PET radiotracer 9-(4-[^18^F]fluoro-3-[hydroxymethyl]butyl)guanine (^18^F-FHBG) has since become the most frequently used tracer for monitoring gene therapy with HSV1-TK and mutant HSV1-sr39TK.^34^ Over the past decade, numerous advancements in HSV1-TK gene therapy have shown preclinical therapeutic success through ^18^F-FHBG PET imaging.^28^^,^^32^^,^^33^^,^^35^^,^^36^ Recent and ongoing studies focus on improving the precision of HSV1-TK gene therapy for specific cancer types or stages.^32^^,^^35^^,^^36^ In line with this objective, Liu et al. employed ^18^F-FHBG PET to evaluate the preclinical therapeutic success of a self-complementing recombinant AAV 3 vector (scrAAV3-HSV1-TK-kallistatin) in targeted liver cancer gene therapy.^32^ Similarly, Kuruppu et al. established a novel therapeutic approach in a preclinical model using conditional oncolytic HSV1 for the targeted treatment of breast cancer leptomeningeal metastases, monitored by bioluminescent imaging and ^18^F-FHBG PET.^36^ However, this study underscores the key limitation that ^18^F-FHBG cannot cross the intact blood-brain barrier (BBB) and can only track HSV1-TK-expressing brain tumors immediately after viral injection, when the BBB remains compromised due to significant tumor burden. As a result, these images may reflect BBB disruption at different stages of tumor progression rather than directly capturing HSV1-TK expression. Despite promising preclinical applications, there are limited clinical studies evaluating the safety and efficacy of *in vivo* HSV1-TK gene therapy. A phase I clinical trial by Sangro et al. demonstrated the safety of intratumoral administration of HSV1-TK gene therapy in patients with hepatocellular carcinoma.^29^ However, the study offered limited insight into the therapy’s efficacy as monitored by ^18^F-FHBG. There was no quantification of HSV1-TK activity, and the injected dose was only detectable in patients who received a dose of ≥10^12^ viral particles, with no dose-expression relationship. Additionally, PET imaging failed to detect transgene expression following a second dose, likely due to the pre-existing immune response against the adenovirus and HSV1-TK^45^ from the initial dose. This immunogenicity could present a significant limitation in the clinical application of HSV1-TK gene therapy. While other studies have safely employed ^18^F-FHBG as an imaging probe in human patients,^46^^,^^47^ these did not directly involve monitoring of HSV1-TK gene therapy. Nonetheless, preclinical findings underscore the potential of HSV1-TK as a dual-purpose tool with both therapeutic and diagnostic applications in cancer gene therapy, highlighting the need for further clinical evaluation.

#### NIS as a theranostic imaging reporter gene

The sodium iodide symporter is another theranostic imaging reporter that has recently demonstrated significant potential in monitoring gene delivery and gene therapy outcomes for cancer, particularly in radionuclide or radiotargeted gene therapy.^20^^,^^27^^,^^48^^,^^49^^,^^50^^,^^51^^,^^52^^,^^53^^,^^54^^,^^55^^,^^56^^,^^57^^,^^58^ The mechanism of NIS gene therapy involves transfer of the NIS gene to tumors using *in vivo* or *ex vivo* methods, enabling the transport of therapeutically active radionuclides, such as ^131^I, ^188^Re, or ^211^At into the tumor cells.^20^^,^^50^ This process also facilitates the uptake of radiolabeled substrates by living tumor cells for PET or SPECT imaging, allowing direct monitoring of the gene therapy outcome at active tumor sites.^20^^,^^49^^,^^51^^,^^52^^,^^53^^,^^56^^,^^57^^,^^58^

Recent and ongoing studies focus on the translation of NIS gene therapy from preclinical studies to human patients through the improvement of gene delivery.^20^^,^^51^^,^^53^^,^^54^^,^^55^ In pursuit of this goal, Spitzweg et al. at Ludwig Maximilians University in Munich recently advanced the use of targeted polyplexes and stem cells as alternatives to viral NIS gene delivery.^20^^,^^51^^,^^55^ In 2021, Spellerberg et al. introduced a novel epidermal growth factor receptor-targeted polyplex-mediated NIS gene therapy for glioblastoma.^55^ This approach employs the therapeutic radionuclide ^131^I and incorporates ^124^I PET imaging to non-invasively monitor the therapy’s effectiveness *in vivo*. Various radionuclides, including ^123^I, ^125^I, ^131^I, ^99m^Tc, and ^188^Re, can also be used to evaluate NIS gene therapy in tumors with SPECT imaging.^59^^,^^60^^,^^61^^,^^62^^,^^63^^,^^64^ Recent advancements have enabled PET imaging with ^18^F-tetrafluoroborate (^18^F-TFB), in addition to ^124^I, in both preclinical and clinical settings, offering improved resolution and sensitivity compared to SPECT radionuclides.^51^^,^^53^^,^^56^^,^^57^^,^^58^^,^^65^^,^^66^^,^^67^ The novel PET tracer ^18^F-TFB has demonstrated the most significant potential for clinical monitoring of NIS gene therapy due to its relatively short half-life for patient safety and lower positron energy for higher image resolution compared to other tracers.^65^ Compared to pharmacodynamic PET tracers like ^18^F-FDG, ^18^F-TFB is more effective at detecting small metastases because it lacks the non-specific uptake seen with ^18^F-FDG in adjacent metabolically active tissues. This advantage was demonstrated by Diocou et al. using an orthotopic xenograft breast cancer model expressing NIS.^67^ An additional benefit of ^18^F-TFB, unlike ^124^I, is that it is not organified by the thyroid (similar to ^**99**m^Tc), allowing it to effectively target non-thyroidal tissues.^20^^,^^56^^,^^66^^,^^68^ In an initial clinical study, O’Doherty et al. demonstrated the safety of ^18^F-TFB for imaging the human NIS in patients with thyroid cancer, showing a biodistribution pattern comparable to ^**99**m^Tc-pertechnetate.^66^ A follow-up comparative study by Samnick et al. further confirmed the clinical utility of ^18^F-TFB PET for monitoring NIS gene therapy in thyroid cancer patients, concluding that this imaging agent performs comparably to the well-established ^124^I-NaI PET.^56^ Subsequent clinical studies by Dittmann et al. and Ventura et al. have further demonstrated the superiority of ^18^F-TFB PET over ^131^I SPECT.^57^^,^^58^ Although ^18^F-TFB PET imaging has been examined in a limited number of human patients, these recent studies indicate that ^18^F-TFB holds significant potential for clinical translation.

#### eDHFR as an emerging imaging reporter gene

Despite the advantages of HSV1-TK and NIS as imaging reporter genes, limitations remain in their applications due to issues such as size constraints, off-target effects, immunogenicity (HSV1-TK), and insufficient radioisotope trapping (NIS).^69^^,^^70^ With the goal to overcome these challenges, *Escherichia coli* dihydrofolate reductase (eDHFR) has been developed as an alternative imaging reporter gene,^71^ with its paired PET probes ^18^C-trimethoprim (TMP)^71^ and ^18^F-TMP^72^^,^^73^ derived from a clinical antimicrobial agent. eDHFR catalyzes the reduction of dihydrofolate to tetrahydrofolate, an essential metabolic process in *E. coli*. Importantly, eDHFR has a unique binding site that differs significantly from the mammalian counterparts, resulting in a 3- to 4-fold higher binding affinity for TMP, making it highly specific and sensitive.^74^ In addition, due to the small size of eDHFR (18 kDa), it may present fewer immunogenic epitopes, though further testing is required. Given the high *in vivo* detection sensitivity (less than a million eDHFR-expressing cells)^71^ and the PET probes derived from clinically approved antibiotics, eDHFR, with its paired PET probes, offers a promising imaging biomarker for monitoring *in vivo* gene therapies.

### Imaging pharmacodynamic biomarkers for cancer gene therapy outcomes

In cancer imaging, pharmacodynamic imaging biomarkers assess specific aspects of tumor metabolism in response to diverse cancer therapy modalities.^17^ Among metabolic radiotracers, ^18^F-FDG is the most widely adopted in cancer imaging.^17^^,^^75^ ^18^F-FDG functions by quantifying the pharmacodynamic biomarker glycolysis, which is increased in living cancer cells due to the Warburg effect.^76^^,^^77^ In cancer gene therapy, ^18^F-FDG can be employed alongside therapeutic tracers to monitor gene delivery and assess efficacy, allowing for comprehensive monitoring of the gene therapy outcome.^31^^,^^57^^,^^78^ However, recent studies have revealed certain shortcomings of ^18^F-FDG alone in monitoring tumor response to gene therapy when compared to more specific radiotracers such as ^18^F-FHBG^33^ and ^18^F-TFB^67^ discussed above. Other metabolic radiotracers, such as ^18^F-FET and 3′-deoxy-3′-fluorothymidine (^18^F-FLT), have shown promise in monitoring treatment and tumor progression in certain cancers, although they have not been studied extensively for gene therapy outcomes.^18^F-FET targets L-amino acid transporters, which are overexpressed in glioma cells, and ^18^F-FLT serves as an indicator of cell proliferation. ^18^F-FET has proven highly effective in assessing glioma tumor metabolic activity and has recently seen significant clinical use for PET imaging in glioma patients.^75^^,^^79^^,^^80^^,^^81^^,^^82^ ^18^F-FLT has demonstrated some success in preclinical studies for monitoring early response to therapy and predicting tumor progression in cancer cells.^83^^,^^84^^,^^85^^,^^86^ Given their success as metabolic radiotracers in cancer imaging, ^18^F-FET and ^18^F-FLT hold significant potential for monitoring gene therapy outcomes in cancer.

## Translational imaging of gene therapy outcomes in metabolic disorders

### Imaging pharmacodynamic biomarkers for gene therapy outcomes in inherited metabolic disorders

Inherited metabolic disorders (IMDs) encompass hundreds of rare conditions that disrupt metabolism, many of which are prime candidates for gene therapy due to their generally well-characterized single-gene origins. Among these, glycogen storage disorders (GSDs) and lysosomal storage disorders (LSDs) have recently garnered significant attention for their successful gene therapy treatments. To support these advancements, translational imaging techniques are used in the IMD studies for effective monitoring of treatment outcomes. A summary of applications of translational imaging in gene therapy to treat IMDs is included in Table 2.

**Table 2:** Application of translational imaging in gene therapy to treat metabolic disorders

Disease	Biomarker	Disorder(s)	Imaging modality	References
GSDs	hepatic adenomas	GSD I	Gd-EOB-DTPA enhanced, T1W, T2W MRI	Li et al.^95^; Vernuccio et al.^96^; Cho et al.^98^
	muscular atrophy	GSD II	T1W MRI	Del Gaizo et al.^100^
		GSD IV	T2W, FLAIR MRI	Mochel et al.^101^
	muscular fat infiltration	GSD II	T1W MRI	Del Gaizo et al.^100^
		GSD III	T1W MRI	Paschall et al.^99^
		GSD IV	T2W, FLAIR MRI	Mochel et al.^101^
	glycogen storage	GSD III	T2W glycoNOE MRI	Zhou et al.^104^; Zeng et al.^105^
	glycolysis	GSD I	^18^F-FDG PET	Sato et al.^107^
LSDs	white matter abnormalities	FD	T1W MRI, FLAIR MRI, SWI MRI, T2W MRI	Lyndon et al.^113^; Sawada et al.^115^; Kono et al.^116^; Cocozza et al.^117^
		metachromatic leukodystrophy	T1W, FLAIR MRI	Biffi et al.^114^
		Hurler syndrome	T2W, FLAIR MRI	Gentner et al.^118^
	cerebral microbleeds	FD	T1W MRI, FLAIR MRI, SWI MRI	Sawada et al.^115^; Kono et al.^116^; Cocozza et al.^117^
	PVSs	FD	T2W, PSIR MRI	Lyndon et al.^113^
		Hurler syndrome	T2W, FLAIR MRI	Gentner et al.^118^
	liver and spleen fibrosis	Gaucher disease	T1W MRI	Razek et al.^121^
	abnormal bone marrow infiltration	Gaucher disease	T1W, TW2 MRI	Andrade-Campos et al.^123^
Other IMDs	cerebral glucose metabolism	GAMT deficiency	^18^F-FDG PET	Khoja et al.^125^
	glutathione synthesis	ASL deficiency	^18^F-FSPG PET	Gurung et al.^129^

#### Gene therapy and imaging for GSDs

GSDs are a group of IMDs (i.e., GSD I, Pompe disease, GSD III, etc.) characterized by a deficiency of enzymes essential for the storage, synthesis, or utilization of glycogen. Gene therapy has been explored for various GSDs, primarily using AAV vector-mediated techniques for restoration of enzymes involved in the formation or breakdown of glycogen.^87^^,^^88^^,^^89^^,^^90^^,^^91^^,^^92^^,^^93^^,^^94^ Historically, translational imaging studies for GSDs have utilized T1-weighted (T1W) or T2-weighted (T2W) MRI to visualize hepatic adenomas, which are common in these disorders.^95^^,^^96^^,^^97^ In 2019, Cho et al. applied T2W MRI to monitor the adenoma-developing stage in GSD Ia before recombinant AAV (rAAV) vector-mediated G6PC gene transfer in mice.^98^ This study determined that rAAV-mediated gene therapy prevents *de novo* hepatocellular adenoma/carcinoma in mice during the adenoma development stage.^98^ Further, diagnostic and disease monitoring studies in patients have used MRI to image fatty infiltration and atrophy in muscles as pharmacodynamic biomarkers correlating with the severity of GSDs.^99^^,^^100^^,^^101^ Although these studies do not directly involve gene therapy, biomarkers used in these studies have potential to monitor GSDs throughout gene therapy treatment in clinical settings.

An ideal pharmacodynamic biomarker for monitoring the treatment of GSD is glycogen storage, which has recently been detected successfully by MRI. Researchers at the Kennedy Krieger Institute first demonstrated that glycogen levels could be assessed through the water signal in MRI using chemical exchange saturation transfer imaging.^102^^,^^103^ Building on this, Zhou et al. introduced a novel method known as glycogen nuclear Overhauser effect (glycoNOE) MRI for *in vivo* glycogen quantification,^104^ and Zeng et al. confirmed its success as a noninvasive imaging method for monitoring GSD III in a mouse model.^105^ PET imaging has also been evaluated to monitor GSD pharmacodynamic biomarkers, yielding mixed results. Plockinger et al. investigated ^18^F-FDG as a potential PET imaging tracer for Pompe disease and found that this radiotracer does not correlate with glycogen storage *in vivo*, suggesting that it may not be an accurate tracer for Pompe disease.^106^ Conversely, a recent case study by Sato et al. suggested that high ^18^F-FDG accumulation in the liver of a patient with GSD I may result from a deficiency of glucose 6-phosphate activity in hepatocytes, highlighting the potential of ^18^F-FDG PET for monitoring GSD I.^107^ However, the study’s findings are limited by its sample size of just one patient. Despite the need for further studies to validate some of these findings, such advancements in imaging methods have significantly enhanced diagnostic capabilities for GSDs and will be essential for monitoring therapeutic outcomes as gene therapies continue to advance.

#### Gene therapy and imaging of LSDs

**Figure 2:**
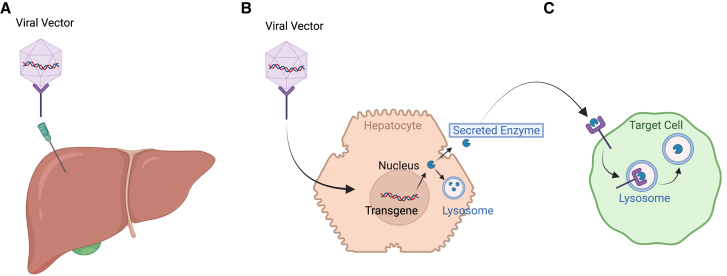
Systemic cross-correction (A) The liver is infected with a viral vector. (B) The virus delivers its genetic materials into the nucleus of hepatocytes. The hepatocytes will produce the enzyme, which will be packaged into lysosomes or secreted into the bloodstream. (C) The enzyme travels through the bloodstream until it reaches the targeted tissue. Once at the target cell, the enzyme binds to the receptor and is trafficked to the lysosome.

LSDs are a group of IMDs resulting from deficiencies in lysosomal enzyme activity. Gene therapy studies have recently employed lentivirus vector and AAV vector gene therapies to deliver therapeutic genes that enhance the expression of specific lysosomal enzymes in target organs.^108^^,^^109^^,^^110^^,^^111^^,^^112^ Compared to other genetic diseases, LSDs have shown considerable success as targets of gene therapy, largely due to a distinctive feature known as “systemic cross-correction.” This trait enables cells in target organs to secrete enzymes into the bloodstream, which then correct enzyme deficiencies in other cells (Figure 2).^108^

MRI serves as a critical tool in diagnosing, monitoring progression, and evaluating treatment outcomes across many LSDs, including Fabry disease (FD), metachromatic leukodystrophy, and Hurler syndrome. Historically, white matter abnormalities and cerebral microbleeds detectable by MRI have been significant pharmacodynamic biomarkers for many LSDs.^113^^,^^114^^,^^115^^,^^116^^,^^117^ A breakthrough study by Lyndon et al. revealed that MRI-visible brain perivascular spaces (PVS) are consistently associated with Fabry disease, suggesting that PVS could serve as another promising pharmacodynamic imaging biomarker for LSDs.^113^ This finding was further confirmed by a clinical lentiviral vector-based hematopoietic stem cell gene therapy study for Hurler syndrome conducted by Gentner et al., who used MRI at baseline and post treatment to demonstrate that the therapy was associated with reduced white matter, cerebral microbleeds, and PVS abnormalities.^118^ Building on these insights, subsequent studies of various LSDs have incorporated PVSs alongside traditional imaging biomarkers to comprehensively monitor the therapeutic outcomes of gene therapies.^115^^,^^119^

Gaucher disease, the most common LSD, has benefitted from recent advancements in gene therapy using AAVs as viable alternatives to traditional enzyme replacement therapy (ERT).^120^ MRI has been pivotal in monitoring treatment outcomes in ERT for Gaucher disease, identifying pharmacodynamic biomarkers such as liver and spleen fibrosis and lesions^121^^,^^122^ and abnormal bone marrow infiltration,^122^^,^^123^ described previously in a review by Degnan et al..^124^ These imaging methods are expected to play a similar role in assessing therapeutic outcomes in gene therapy for Gaucher disease.

#### Gene therapy and imaging of other IMDs

Other IMDs have shown significant potential for gene therapy. However, research on translational imaging biomarkers for these disorders is limited and remains a subject for further exploration. Nevertheless, notable recent studies are included in this section.

Guanidinoacetate methyltransferase (GAMT) deficiency is a rare creatine deficiency disorder that has recently seen significant potential for AAV-based hepatic gene therapy.^125^^,^^126^ In GAMT deficiency, the near-complete absence of creatine in the brain is associated with neuronal dysfunction. To overcome the limitations of previous treatments for GAMT deficiency, Khoja et al.. (2022) introduced a novel gene therapy approach to express human codon-optimized GAMT in hepatocytes. This study employed ^18^F-FDG PET imaging to measure cerebral glucose metabolism as a quantitative pharmacodynamic biomarker for neuronal dysfunction in GAMT-deficient and GAMT-restored mice, determining that CNS function was restored after gene therapy.^125^ This imaging method has the potential for imaging gene therapy outcomes in other IMDs involving neuronal dysfunction and dysregulation of glucose metabolism.

Arginosuccinate lyase (ASL) deficiency is a urea cycle disorder that has gained recent attention for viral and nonviral gene therapies.^127^^,^^128^^,^^129^ One determining characteristic of this disorder is the dysregulation of hepatic glutathione biosynthesis, resulting in liver disease. Gurung et al.. reported (4S)-4-(3-^18^F-fluoropropyl)-L-glutamic acid (^18^F-FSPG) as a PET radiotracer for hepatic glutathione synthesis in ASL deficiency to monitor the extent of disease and treatment response in mice. Although this study employed mRNA therapy by *ex vivo* methods to correct disease symptoms, the authors argued that the use of ^18^F-FSPG PET imaging in assessing therapeutic outcomes in ASL would not be dependent on the therapeutic method used; therefore, this approach may be applied to *in vivo* gene therapy using viral vectors, non-viral vectors, and gene editing.^129^

## Imaging of gene therapy outcomes in tissue degenerative disorders

### Neurodegenerative disorders

**Figure 3:**
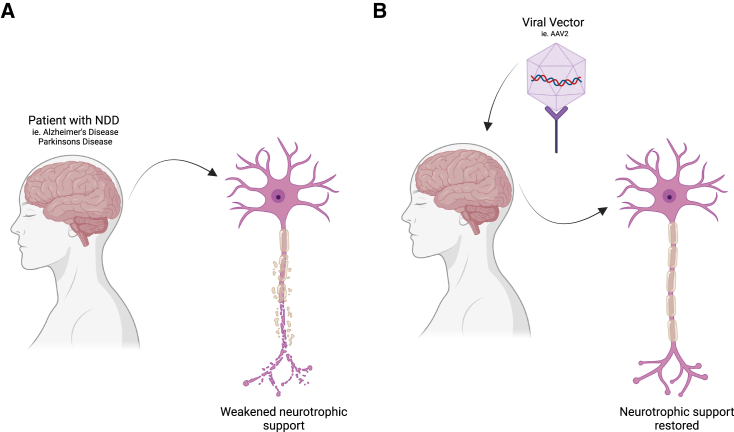
Gene therapy for NDDs (A) A patient with NDD exhibiting weakened neurotrophic support. (B) Introduction of a viral vector into a neuronal cell restores neurotrophic support by delivering genetic material (i.e., AAVs or neurotrophic factors).

Neurodegenerative disorders (NDDs), such as Alzheimer’s disease (AD) and Parkinson’s disease (PD), are biologically heterogeneous disorders affecting the CNS, causing cognitive and motor impairment due to neuronal death.^130^ While traditional pharmacological interventions for NDDs have faced many setbacks, gene therapy has emerged as a promising approach to address the root causes.^131^^,^^132^ In this section, we use AD and PD as two examples that utilize molecular imaging to track gene therapy outcomes (Figure 3). A summary of recent applications of translational imaging in gene therapy to treat NDDs is included in Table 3.

**Table 3:** Application of translational imaging in gene therapy to treat tissue-degenerative disorders

Disease	Biomarker	Imaging modality	References
AD	glycolysis	^18^F-FDG PET	Rafii et al.^144^; de Bellis et al.^148^; Carbonell et al.^157^
	amyloid and tau burden	^18^F-florbetaben, ^18^F-florbetapir, ^18^F-flutemetamol, ^11^C-PiB, ^18^F-flutafuranol PET	Iwata et al.^161^; Duro et al.^162^; Wu et al.^163^; Papadakis et al.^164^; Nunes Schuck et al.^165^; Li et al.^166^; Oh et al.^167^
	cognitive performance	fMRI	Tripoliti et al.^168^; Leal et al.^170^
	functional connectivity abnormalities	resting-state fMRI	Dickerson et al.^171^; Schultz et al.^172^
	white matter abnormalities	diffusion MRI	Bozzali et al.^174^; Bennett et al.^175^
	cerebral atrophy	structural MRI	Raz et al.^176^; Yassa et al.^177^; Ewers et al.^178^
PD	dopaminergic metabolism	^18^F-FMT PET, ^18^F-DOPA PET	Palfi et al.^8^; Bankiewicz et al.^182^; Mittermeyer et al.^184^; Heiss et al.^189^; Whone et al.^190^; Tai et al.^191^
	synaptic dopamine levels	^11^C-raclopride PET	Palfi et al.^8^
	glycolysis	^18^F-FDG PET	Niethammer et al.^186^
Osteoporosis	bone mass	microCT	Yang et al.^194^; Yang et al.^195^; Oh et al.^196^; Lin et al.^197^
	connectivity density	microCT	Yang et al.^194^; Oh et al.^196^

#### Gene therapy and imaging of AD

The neuropathology of AD is characterized by misfolded protein accumulation, primarily hyperphosphorylated tau and β-amyloid (Aβ).^133^^,^^134^^,^^135^ In addition, key factors in AD pathogenesis include oxidative stress, impaired glucose metabolism, and heightened neuroinflammation, all of which interact with amyloid pathology.^135^^,^^136^^,^^137^ Advances in imaging biomarkers for these key factors in AD pathogenesis over the past decade have proven advantageous in monitoring gene therapy outcomes.^138^

One gene therapy method that has recently shown success for treatment of AD is AAV2-neurotrophic factor (NGF) gene therapy. While oral dosing of NGF is ineffective due to its inability to cross the BBB,^139^^,^^140^^,^^141^ AAV2-NGF gene therapy has shown safety and long-term transgene expression.^142^^,^^143^ In a recent clinical study, to monitor therapeutic outcomes, Rafii et al. used ^18^F-FDG PET imaging to assess brain metabolic activity in AD patients at baseline and 6, 12, and 24 months post AAV2-NGF therapy.^144^ The study found a progressive increase in brain ^18^F-FDG PET signal intensity in the treatment group 24 months post therapy.^144^^,^^145^^,^^146^^,^^147^ Similar results were achieved by de Bellis et al., who observed increased ^18^F-FDG uptake in the various brain regions 1 year post therapy.^148^ Another example of AD gene therapy involves targeting three different apolipoprotein E (ApoE) polymorphic alleles. The ε4 allele is the primary risk factor for late-onset AD, while the ε2 allele is protective.^149^^,^^150^ Jackson et al. found that AAV1-ε2 gene therapy reduced brain Aβ plaque deposition, synaptic loss, and microglial activation in ApoE4-TR/APP/PS1 mice (APP, amyloid precursor protein; PS1, presenilin 1).^151^ Previous studies have shown that young ε4 carriers exhibit cerebral glucose hypometabolism.^152^^,^^153^^,^^154^^,^^155^^,^^156^ By using ^18^F-FDG PET imaging, researchers like Carbonell et al. demonstrated a strong association between ε4, Aβ, and glucose hypometabolism in early AD stages,^157^ suggesting ^18^F-FDG PET as a useful pharmacodynamic imaging biomarker for AD gene therapy outcomes.^158^

Amyloid burden imaging provides another pharmacodynamic biomarker to monitor AD gene therapy outcomes, with the PET tracers ^18^F-florbetaben, ^18^F-florbetapir, and ^18^F-flutemetamol (US Food and Drug Administration approved) and ^11^C-PiB and ^18^F-flutafuranol (research purposes).^159^^,^^160^ For example, Iwata et al. used ^11^C-PiB microPET to monitor neprilysin gene therapy in APP transgenic mice.^161^ Development of more sensitive and selective imaging tracers for amyloid and tau detection continues to be a promising area of AD research,^162^^,^^163^^,^^164^^,^^165^^,^^166^^,^^167^ improving our abilities to monitor AD gene therapy outcomes.

MRI is also used in AD gene therapy studies to determine the therapeutic outcomes with its capacity to identify early AD pharmacodynamic biomarkers.^138^ Specifically, functional MRI (fMRI) indirectly measures neuronal activity by detecting local changes in blood magnetic properties related to brain metabolism.^138^^,^^168^^,^^169^ Other MRI techniques, such as task-activated fMRI, resting-state fMRI, diffusion MRI, and structural MRI, are used to confirm disease presence or structural changes in AD.^170^^,^^171^^,^^172^^,^^173^^,^^174^^,^^175^^,^^176^^,^^177^^,^^178^ While structural MRI and ^18^F-FDG PET were once thought to be interchangeable for measuring AD burden, recent data suggest that they provide complementary information.^138^ Benvenutto et al. found that ^18^F-FDG PET is better for tracking clinical severity, while structural MRI markers are more associated with cognitive reserve.^179^ These findings highlight the importance of selecting the appropriate imaging biomarker to determine gene therapy outcome.

#### Gene therapy and imaging of PD

PD is characterized by the loss of dopaminergic neurons in the substantia nigra pars compacta and reduced dopamine levels in the striatum.^131^ Therefore, ideal pharmacodynamic imaging biomarkers for PD therapies include dopamine and dopaminergic metabolism, monitored by PET radiotracers. The current standard treatment, dopamine replacement therapy, does not delay disease progression and has numerous side effects.^180^ Targeted gene therapy for PD are emerging, categorized as disease-modifying and non-disease modifying.^180^

Non-disease modifying treatments aim to reduce PD symptoms by expressing dopaminergic or GABAgenic enzymes to normalize basal ganglion activity. Studies using striatal vector-mediated aromatic L-amino acid decarboxylase (AADC) overexpression gene therapy in non-human primates (NHPs) showed reduced parkinsonian symptoms.^181^^,^^182^^,^^183^ The PET radiotracer ^18^F-fluoro-meta-tyrosine (FMT) has been employed to trace dopaminergic activity, serving as a pharmacodynamic biomarker for monitoring outcomes of AADC overexpression gene therapy in both NHPs and human patients. For instance, Bankiewicz et al. used ^18^F-FMT PET to confirm transgene expression of AAV2-AADC in NHPs by monitoring dopaminergic activity before and after gene transfer.^182^ In a phase I clinical trial of AADC gene therapy for PD, Mittermeyer et al. similarly used ^18^F-FMT PET to monitor patients with moderate PD who received bilateral putaminal infusions of the AAV2-hAADC vector.^184^ The trial results showed that the ^18^F-FMT PET signal increased in these patients 12 months post treatment and was sustained over a 4-year period, indicating prolonged stabilization of transgene expression.^184^ PET imaging is also used to monitor the lentiviral vector gene therapy ProSavin, another non-disease modifying treatment. In a dose escalation clinical study,^8^ ^18^F-levodopa and ^11^C-raclopride PET imaging was conducted on the day of dose administration and 6 months post ProSavin dosing.^8^ While ^18^F-levodopa PET did not show significant changes, ^11^C-raclopride PET imaging showed a significant dose effect in dopamine putaminal binding potential. As a selective competitive D_2_/D_3_ antagonist, ^11^C-raclopride binds to striatal dopamine receptors to monitor changes in synaptic dopamine levels,^185^ an additional pharmacodynamic biomarker for PD. ^18^F-FDG PET imaging has also been used to study gene therapy outcomes for non-disease-modifying treatments of PD. In a clinical study, Niethammer et al. used ^18^F-FDG PET to monitor the outcome of bilateral delivery of the glutamic acid decarboxylase (GAD) gene into the subthalamic nuclei (STN) of advanced PD patients.^186^ Glucose metabolism served as a pharmacodynamic biomarker to evaluate the therapeutic outcome of AAV2-GAD gene therapy, revealing a trend of decreased brain glucose metabolism in the AAV2-GAD treatment group.

Disease-modifying gene therapies for PD have also been studied extensively, with molecular imaging playing an important role in monitoring treatment efficacy. Growth factors, particularly the glial cell line-derived neurotrophic factor family of ligands, including glial cell line-derived neurotrophic factor (GDNF), neurturin, artemin, and persephin, have been popular treatment targets.^8^^,^^187^^,^^188^ Heiss et al. studied the first-in-human use of the AAV2-GDNF vector co-infused with gadoteridol via convection-enhanced delivery into the bilateral putamina. T1W MRI tracked AAV2-GDNF infusion, while ^18^F-fluorodopa (^18^F-DOPA) PET measured the course of the disease.^189^ The results showed that ^18^F-DOPA PET could monitor gene therapy outcomes over 18 months, evident by GDNF expression though enhanced ^18^F-DOPA neuronal uptake.^189^ ^18^F-DOPA PET has subsequently been used in multiple clinical trials to monitor dopaminergic metabolism as a pharmacodynamic biomarker for PD, providing insights into gene transduction durability.^190^^,^^191^

### Bone-degenerative disorders

Bone-degenerative disorders encompass a diverse range of conditions affecting bones and joints. Gene therapy has been explored as a potential treatment for regenerating tissues and systems impacted by these disorders.^192^^,^^193^^,^^194^^,^^195^^,^^196^^,^^197^ CT is currently the predominant method used to monitor therapeutic outcomes in gene therapy studies for tissue regeneration in skeletal disorders. A summary of recent applications of translational imaging in gene therapy to treat bone-degenerative disorders is included in Table 3.

#### Gene therapy and imaging of osteoporosis

Osteoporosis is a degenerative skeletal disorder characterized by bone weakening, most common in the elderly. This condition has recently gained attention as a candidate for *in vivo* virus-based gene therapy, following groundbreaking preclinical studies by Shim and colleagues.^194^^,^^195^^,^^196^^,^^197^ Studies conducted in 2019 and 2020 were the first to identify that silencing the expression of proteins such as Schnurri-3 (SHN3)^195^ and cathepsin K^194^ via bone-targeting rAAV9 could be effective approaches for treating osteoporosis. A subsequent study by Oh et al. (2023) expanded on this work, demonstrating that a bone-targeted rAAV carrying artificial microRNAs could silence the expression of WNT antagonists, SHN3, and sclerostin, thereby enhancing WNT/β-catenin signaling, osteoblast function, and bone formation in a mouse model.^196^ More recently, a 2024 study by Lin et al. applied gene therapy targeting SHN3 to treat alveolar bone loss in a mouse model of osteoporosis.^197^ All of these studies utilized microCT to monitor the therapeutic outcomes of gene therapy by examining bone microarchitecture characteristics as pharmacodynamic biomarkers for successful treatment. The most common indicator of osteoporosis is bone mass loss, which microCT quantifies using parameters such as bone thickness and bone volume fraction (the ratio of bone volume to total volume). In all mentioned studies conducted by Shim and colleagues, bone mass increased following gene therapy, demonstrating its efficacy in bone regeneration. Another biomarker employed in some studies was connectivity density, a measurable indicator of connectivity between fracture ends resulting from augmented bone formation post gene therapy.^194^^,^^196^ These CT biomarkers will continue to be integral in diagnosing and monitoring treatment outcomes in skeletal diseases. Although gene therapy for skeletal disorders is still in its early stages, continued reliance on CT imaging is anticipated for monitoring the outcomes of future advancements in gene therapy-mediated tissue regeneration.

## Prospects for incorporating imaging biomarkers into the development and application of gene therapy

Although gene therapy has made significant progress and offers long-term treatment solutions for an expanding range of diseases, several challenges persist, including targeting the wrong cells and tissues, host immune responses, and loss of transgene expression.^198^ The incorporation of imaging biomarkers into gene therapy has the potential to address and mitigate many of these enduring issues.

### Imaging gene delivery

Strategies have been developed to label viral capsids with positron emitting radionuclides for PET imaging, enabling real-time tracking of gene delivery. Two primary approaches for labeling AAV capsids have emerged. One involves labeling with I-124 using direct (Iodogen) or indirect (modified Bolton-Hunter) methods,^199^^,^^200^ while the other is Cu-64 labeling via a multichelator structure (NOTA)_8_.^13^ While ^64^Cu-(NOTA)_8_ adds relatively large molecular constructs to the AAV capsid, I-124 can be bound directly, ensuring minimal structural alterations. The I-124-capsid labeling approach has been validated extensively for *in vivo* AAV capsid imaging across multiple species, including rodents and NHPs.^199^^,^^200^ On the other hand, the ^64^Cu-(NOTA)_8_ method, by binding more radionuclide atoms per AAV capsid, shows promise for imaging AAV biodistribution in the brain but needs further validation in species other than rodents.^13^^,^^201^ Each method offers distinct advantages depending on the specific application. A detailed comparison of these two AAV capsid labeling methods is presented in Table 4. Furthermore, a recent study demonstrated the quantification of both biodistribution and transduction of systemically administered AAV labeled with ^64^Cu-(NOTA)_8_ on the capsid, which also carried a PET reporter gene.^202^ This strategy provides a valuable platform for assessing the pharmacokinetics, cellular targeting, and protein expression kinetics of AAV vectors in gene therapy applications.

**Table 4:** AAV capsid labeling with ^124^I and ^64^Cu-(NOTA)_8_

	I-124 labeling		^64^Cu-(NOTA)_8_
	Iodogen method	Modified Bolton-Hunter method	
Added structure size per labeling site	124 Da	371 Da	5,000–6,000 Da
Infectivity of labeled AAV	72% of control	63% of control	comparable to control
Radionuclide atoms per capsid	13.36	3.17	18–28
Half-life of radionuclide	4.18 days		12.7 h
Positron fraction	22.5%		17.9%
Maximum positron energy	2.14 MeV		0.653 MeV
Mean positron range in water	3.5 mm		0.56 mm
PET image spatial resolution (full width at half-maximum)^210^	3.31 mm		2.44 mm
Species tested	mice,^199^ monkeys^200^		mice^13^^,^^201^

### Imaging gene activation

Many *in vivo* gene therapy approaches are designed as “one and done” treatments, but accumulating evidence indicates that re-dosing may be necessary due to a decline in therapeutic gene activation. The duration of transgene activation can be limited by the lifespan of the target cells or by the host’s immune response to the transgene. For instance, in a phase III trial of valoctocogene roxaparvovec, which uses AAV5 to deliver a factor VIII cDNA under regulation of a liver-specific promoter for treating hemophilia A, the mean transgene activation decreased from 45 IU/dL at 8 months to 25 IU/dL by the end of year 2.^203^ Incorporating imaging reporter genes into gene therapy could facilitate non-invasive monitoring of transgene activation and help determine the need for re-dosing if this becomes a therapeutic option in the future. The choice of the reporter should not interfere with the function of the therapy. For example, imaging reporter genes derived from human genes could minimize immune responses, such as NIS for [^18^F]-TFB PET imaging,^52^ and oatp1a for In-111 SPECT imaging and gadolinium-ethoxybenzyl-DTPA contrast T1W MRI.^204^ While human reporter genes may have the least potential for immunogenicity, as was found with immune responses to HSV-TK,^45^ they are likely to have high background and variable expression in tumors, which may confound their use as a therapeutic biomarker. New, engineered proteins, such as membrane-bound single-chain variable fragment for DOTA-haptens labeled with imaging radionuclides,^205^ or intracellular expressed proteins from other species or kingdoms, such as the eDHFR enzyme for ^18^F-TMP PET imaging,^72^^,^^73^ are providing potential tools for imaging transgene activation *in vivo*. In addition, it is important to select reporters for PET or SPECT imaging that use probes labeled with short-lived radioisotopes to minimize the radiation exposure of the patient.

### Imaging liver toxicity

Liver toxicity is a prevalent adverse event associated with AAV-based gene therapy, especially at high doses, due to the sequestration of AAVs in the liver following administration. Liver toxicity results from complement-mediated recognition of the AAV capsid and subsequent CD8^+^ T cell-mediated attacks on hepatocytes that present the capsid on major histocompatibility complex.^198^^,^^206^^,^^207^ Non-invasive imaging biomarkers have been developed for monitoring liver injury and failure, such as ^18^F-2-deoxy-2-fluoroarabinose for PET imaging.^208^ As imaging biomarkers for monitoring liver toxicity are being developed, their integration into gene therapy protocols should facilitate early detection of liver injury and prompt intervention with treatments such as corticosteroids.

### Imaging therapeutic outcomes of current and emerging gene therapies

**Figure 4:**
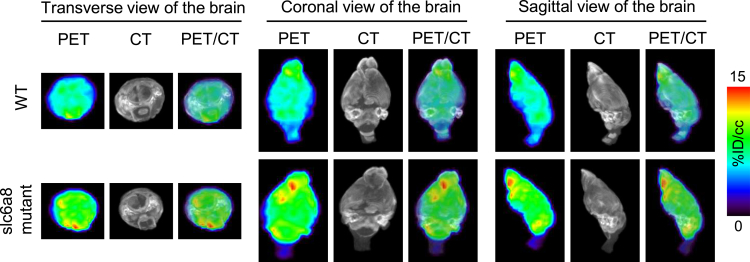
^18^F-FDG PET/CT images of the brain in wild-type and Slc6a8 mutant mice PET/CT images were acquired on the GNEXT PET/CT scanner (Sofie Biosciences) following 60 min of conscious ^18^F-FDG uptake.

In the past decade, a substantial array of imaging biomarkers has been developed for diagnosing various diseases, including cancer, metabolic disorders, tissue-degenerative diseases, cardiovascular diseases, and abdominal diseases.^209^ Furthermore, new imaging biomarkers or applications continue to be developed for diseases that can be treated with current or new gene therapies. For instance, inherited loss-of-function mutations in SLC6A8, which encodes the creatine transporter, result in a cerebral creatine deficiency syndromes (CCDSs) that primarily affect the CNS. We have found that ^18^F-FDG PET imaging reliably and quantitatively diagnoses cerebral creatine deficiency with increased FDG uptake in the brain in a transgenic mouse model of Slc6a8 deficiency (Figure 4), similar to our findings in GAMT deficiency and the associated gene therapy,^125^ offering new tools to support the development of gene therapy for CCDSs. These biomarkers will enable the use of high-quality images to quantitatively and precisely monitor the outcomes of gene therapy in both preclinical and clinical settings.

## Acknowledgments

The authors thank all members of UCLA Crump Preclinical Imaging Technology Center (https://imaging.crump.ucla.edu), particularly Andrea Sarabia, Andrea Litwak, and Sophie Shumilov, for their critical support and Dr. Ikotun (UCLA) for valuable discussions. The work is supported by a NIH Cancer Center support grant (2 P30 CA016042-44), a DISC 2 Quest- Discovery Stage Research Project grant (DISC2-14090 to G.S.L.), a California Institute Regenerate Medicine grant (TRAN1-15227 to G.S.L.), and 10.13039/100000002NIH/10.13039/100000065NINDS (R01NS110596 to G.S.L.). Our PET imaging work in this article was supported by an NIH S10 Shared Instrumentation for Animal Research grant (1 S10 OD026917-01A1) for the GNEXT PET/CT scanner (Sofie Biosciences, Dulles, VA, USA).

## Author contributions

I.L.D., M.T., and S.X. contributed to the writing and review of drafts. G.S.L. contributed to the review of drafts. G.S.L. and S.X. contributed to experiment design of the SLC6A8 imaging biomarker study.

## Declaration of interests

G.S.L. serves as a consultant to Astellas Gene Therapies and has received grant support from the Association of Creatine Deficiencies in an area unrelated to the work described in this manuscript.
